# Three-Dimensional Geometry of Collagenous Tissues by Second Harmonic Polarimetry

**DOI:** 10.1038/s41598-017-02326-7

**Published:** 2017-06-01

**Authors:** Karen Reiser, Patrick Stoller, André Knoesen

**Affiliations:** 1 School of Medicine, University of California, Davis, Department of Neurological Surgery, Davis, CA 95616 USA; 2 Lawrence Livermore National Laboratory, Medical Technology Program, Livermore, CA 94550 USA; 3 ABB Corporate Research, ABB Switzerland Ltd., Baden-Dättwil, Switzerland; 4 College of Engineering, University of California, Davis, Department of Electrical and Computer Engineering, Davis, CA 95616 USA

## Abstract

Collagen is a biological macromolecule capable of second harmonic generation, allowing label-free detection in tissues; in addition, molecular orientation can be determined from the polarization dependence of the second harmonic signal. Previously we reported that in-plane orientation of collagen fibrils could be determined by modulating the polarization angle of the laser during scanning. We have now extended this method so that out-of-plane orientation angles can be determined at the same time, allowing visualization of the 3-dimensional structure of collagenous tissues. This approach offers advantages compared with other methods for determining out-of-plane orientation. First, the orientation angles are directly calculated from the polarimetry data obtained in a single scan, while other reported methods require data from multiple scans, use of iterative optimization methods, application of fitting algorithms, or extensive post-optical processing. Second, our method does not require highly specialized instrumentation, and thus can be adapted for use in almost any nonlinear optical microscopy setup. It is suitable for both basic and clinical applications. We present three-dimensional images of structurally complex collagenous tissues that illustrate the power of such 3-dimensional analyses to reveal the architecture of biological structures.

## Introduction

**Figure 1:**
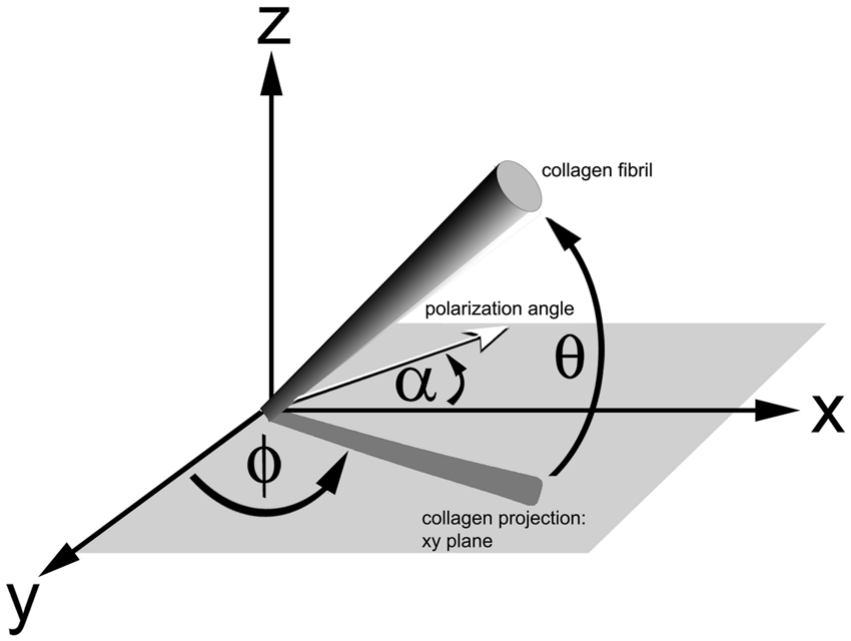
Sketch of collagen fiber orientation in the laboratory frame. The laser beam is incident along the *z* axis and orthogonal to the *xy* plane; *α* refers to the polarization angle of the incident beam with respect to the *x* axis; *ϕ* refers to the angle between the fiber axis in the *xy* plane and the *y* axis; *θ* refers to the angle between the collagen fibril and the *xy* plane.

Second harmonic generation (SHG) is a second order nonlinear optical process that can be induced by intense laser radiation in several biologically important macromolecules, including the polypeptides collagen, tubulin and myosin and the plant polysaccharides amylose and cellulose. SHG imaging offers label-free deep optical sectioning with virtually no background noise. In addition, the polarization-dependence of the SHG signal allows determination of molecular orientation within complex macromolecular arrays. The central role of collagen in the structure and function of virtually every organ system has long stimulated interest in exploiting its nonlinear optical properties to better understand the mechanisms through which it influences behavior of resident cells, and through which it in turn is influenced^1–3^. Geometric cues in particular appear to play a central role in both normal and pathological events, ranging from directing stem cell maturation pathways in bone and cartilage via pore size to initiating neoplastic invasion by remodeling the local matrix structure^4^. Methods for determining collagen orientation in three dimensions rely on polarimetry, essentially analyzing the effects of the input polarization on the intensity of the SHG signal. The overall objective is to measure the collagen fibril orientation, represented by the in-plane angle orientation angle *ϕ* and the out-of-plane angle *θ*, illustrated in Fig. 1, as a function of position from the polarization dependence of the SHG signal. We have previously reported on the use of polarization modulation to measure in-plane angle orientation angle *ϕ*(*x*, *y*) of collagen fibrils^5, 6^. A linear polarized laser beam is focused onto the sample and the angle of polarization is continuously rotated at frequency *ω*. A photodetector picks up the second harmonic signal generated by collagen, and two lock-in amplifiers measure the amplitude and phase of the first and second modulation tones. There are no post-sample polarizing elements. The in-plane orientation angle *ϕ* of the collagen fibrils can be determined from the phase of either the first or second modulation tone. Direct measurement of the out-of-plane angle *θ* has been an elusive goal, however. In this communication we report on methods for determining the out-of-plane angle *θ*(*x*, *y*) from the lock-in data, thus enabling 3-dimensional visualization of fibrillar arrays.

In the last two decades several groups have used second-harmonic polarization imaging to study biological assemblies. For example, instead of continuously varying the linearly polarized input angle, one can use a finite set of linearly polarized input angles and determine the in-plane orientation angle *ϕ* by data-fitting^7^. Recently, Psilodimitrakopoulos *et al*.^8^ have reported on a rapid single-scan approach that uses circular polarization as the input polarization; the SHG signal is split and collected by three polarization analyzers that in effect measure the intensity of three linear polarized states (0°, 45°, 90°). Image acquisition is extremely rapid, largely because the polarization is not modulated, enabling the investigators to obtain data from unanesthetized, moving *C. elegans*. These experimental methods are restricted to in-plane orientation measurements, which may not impose significant limitations on interpretation, given that the myosin filaments under study are almost parallel to the sample plane. Experimental attempts to extract out-of-plane orientation are challenging, and rely on knowing the ratio of second order nonlinear tensor components. Tuer *et al*. have described a method termed ‘polarization-in, polarization-out’ microscopy (PIPO), which is an extension of an approach originally developed by Roth and Freund^3, 9^. A series of small regions (2 × 2 pixels) are repeatedly scanned using multiple combinations of polarizer/analyzer angles. Susceptibility ratios and weighted averages of collagen orientation values are determined by a multistep fitting process of the SHG intensity data sets to a general intensity equation for PIPO SHG to retrieve the out-of-plane angle, as well as an asymmetry parameter related to fibrillar organization. Recently, a new theoretical framework to determine in- and out-of-plane orientation has been proposed that combines SHG polarimetry data with *ab initio* calculations of the predicted molecular nonlinear optical response of a single collagen triple helix^10, 11^. Four commonly used experimental architectures were explicitly explored, including polarization rotation with no post-sample optics, polarization-in polarization-out measurement, and polarization modulation with and without post-sample optics. The approach was applied to a study of several collagenous tissues using nonlinear optical Stokes ellipsometry (NOSE), which is an extension of a technique described in previous papers^12^. NOSE rotates the angle of the input through a finite set of linearly polarized input angles. The polarization of the SHG is analyzed by measuring the vertically and horizontally polarized SHG intensities with two detectors. Values for in-plane and out-of- plane orientation angles are not directly determined from the polarimetry data but are calculated by a series of iterative algorithms. No 3-dimensional reconstructions of the analyzed tissues were reported.

We describe a new method, referred to as SHARP (second harmonic rotating polarization), for analyzing the three dimensional geometry of collagenous arrays in which we derive values for both in-plane and out-of-plane orientation angles directly from SHG polarimetry measurements obtained in a single scan. The SHARP imaging method provides replicability and rapid image acquisition, making it feasible to detect real-time changes in spatial geometry in native tissue. These features are not found in methods that rely on repeated scans using multiple setting of polarizers and analyzers, are restricted to measurement of in-plane fibril orientation, and/or require numerical analysis of the generated signal. Further, for calculations of out-of-plane orientation we use macroscopic susceptibility tensor values, experimentally measured by us using native tissue and confirmed through quartz calibration testing^5, 13^. We thus avoid the persistent problem, described in the studies above, of bridging the gap between molecular hyperpolarizability tensor values and the SHG data obtained at a macroscopic scale. In summary, the speed and versatility of SHARP may prove to be uniquely useful in providing new perspectives on the play of forces that drive both normal matrix processes, such as repair and remodeling, as well as the pathological changes in disease and degeneration.

## Results

### SHARP Imaging

Polarization dependence of the second harmonic intensity of collagen has been extensively discussed in the literature^1, 14, 15^. The electric field of the incident beam $\overrightarrow{E}=\hat{e}{E}_{1}$ is linearly polarized and the angle of the polarization is rotated at a frequency Ω, i.e. $\hat{e}=\,\cos \,\mathrm{(2}\pi {\rm{\Omega }}t)\hat{x}+\,\sin \,\mathrm{(2}\pi {\rm{\Omega }}t)\hat{y}$ for a z-propagating beam. In most such studies, including ours^6, 13^, collagen is assumed to be a cylindrically symmetric distribution of single-axis molecules. It is further assumed that Kleinman symmetry is valid^16^, since all the optical frequencies are much smaller than any molecular resonance frequency in collagen. Under these assumptions, there are only two independent tensor elements in the second-order susceptibility tensor. The induced polarization oscillating at 2*ω* is $\vec{P}(2\omega )={\varepsilon }_{o}a{E}_{1}^{2}\vec{p}$. The director parallel to $\vec{P}(2\omega )$ is $\vec{p}=\hat{s}{(\hat{s}\cdot \hat{e})}^{2}+\gamma \hat{s}(\hat{e}\cdot \hat{e})+2\gamma \hat{e}(\hat{s}\cdot \hat{e})$ where $\hat{s}$ represents the unit vector along the symmetry axis of the collagen fibril and *γ* represents the ratio of the two independent coefficients of the nonlinear susceptibility tensor (*γ* = *b*/*a*). In the coordinate system illustrated in Fig. 1, the expression for the fibril unit vector is: $\hat{s}=\,\cos \,(\theta )\hat{x}+\,\cos \,(\theta )\,\cos \,(\varphi )\hat{y}$. In a coordinate system described by *ijk* with the *k*-axis along the symmetry axis, the two independent *d*–coefficients are *d*_*kkk*_ = (*a* + 3*b*)/2, and *d*_*iik*_ = *b*/2, and *d*_*kkk*_/*d*_*iik*_ = *γ*/(1 + 3﻿*γ﻿*). It can be shown that second harmonic intensity is

1 $${I}_{SHG}\propto {I}_{0}(\gamma ,\theta )+{I}_{1}(\gamma ,\theta )\,\cos \,(2\pi {\rm{\Omega }}t+2\varphi )+{I}_{2}(\gamma ,\theta )\,\cos \,(4\pi {\rm{\Omega }}t+4\varphi )$$

where *θ* represents the out-of-plane orientation angle of the fibril and *ϕ* represents the in-plane orientation of the fibril.

Synchronous detection with a single photodetector and two lock-in amplifiers are used to detect amplitude and phase of the first and second modulation tones. The optical set-up is configured such that, to a good approximation, the second harmonic for each data point is generated in the volume defined by the Gaussian beam spot size and its confocal parameter. The dimensions of this volume, which we will refer to as a voxel, are 1.5 micron in the transverse direction and 10 microns in the axial direction.

There are two approaches for determining the nonlinear tensor ratio of a collagen fibril, one theoretical and the other experimental. The theoretical approach is exemplified in the recent study of Dow *et al*.^11^, in which *ab initio* calculations are used to determine the second order hyperpolarizability tensor elements of a triple helix molecule; the molecular calculations are extended to obtain the hyperpolarizability of a fibril. We and others use an experimental approach to find the ratio *d*_*kkk*_/*d*_*iik*_ or alternatively *γ* ^5, 17, 18^. Experimentally it has been shown that to a good approximation the ratio *γ* is a constant that depends on the genetic type of collagen^5, 18^. Therefore, for studies of tissues comprising fibrillar Type I collagen, *γ* can been measured in any Type I collagen matrix consisting of fibrils that are well- ordered and parallel to a substrate. We have shown, by measurement, that a representative value for *γ* is between −0.70 and −0.60 for Type I collagen, with an average value of −0.65^17^. This general result has been confirmed subsequently by Tiaho *et al*., using a similar measurements approach. For the work reported in this communication, we have confirmed the general conclusions are invariant when values of *γ* are used in the specified range^19^.

An important observation from equation 1 is that the in-plane angle *ϕ* is uniquely determined by the phase of either the first modulation tone or the second modulation tone while the out-of-plane angle *θ* is completely determined by the amplitude terms. In other words, since the in-plane and out-of-plane orientation angles (*ϕ*, *θ*) are determined independently of each other, it is possible to determine average orientations even in structurally complex regions of collagen assemblies. While the ratio of any two of the three amplitudes can be used to obtain information about the out-of-plane angle *θ*, in practice the simplest experimental configuration consists of synchronous detection of the phase and magnitude of the first and second modulation tones using lock-in amplifiers synchronized to modulation frequency Ω. The ratio of the amplitudes of the first and second modulation tones reduces to

2 $$\kappa =-\frac{1}{4}(\frac{{\cos }^{2}\,\theta }{2\gamma +{\cos }^{2}\,\theta })$$

The maximum value of *κ* coincides with the fibril position parallel to the polarization plane and the minimum value corresponds to fibril orientation perpendicular to the polarization plane, i.e. *θ* = *π*/2. When the fibril is parallel to the polarization plane, *κ* is independent of the fibril orientation. If *κ* is smaller than the maximum value, the average fibril orientation may be oriented out of the plane; alternatively, two intersecting fibrils may be present in the voxel, a phenomenon that also reduces the second harmonic intensity. Distinguishing between the effects of fibril intersection and out-of-plane orientation is central to our method.

Intersecting fibrils are most likely to occur at the boundaries between regions with dissimilar collagen orientation^?^. Using sliding neighborhood analysis methods, we identify boundary voxels with intersecting fibrils and calculate their acute intersection angle, *δϕ*; see **Methods** online. If we assume that the confocal length is approximately equal to the coherence length of collagen (typically on the order of 7 microns)^13^, then for the intersecting fibril case, the ratio of amplitudes of the first and second modulation tones is

3 $${\kappa }_{i}=\frac{{I}_{2}(\gamma ,\theta )}{{I}_{1}(\gamma ,\theta )}=-\frac{1}{4}\frac{\cos \,(2\delta \varphi )}{\cos \,(\delta \varphi )}(\frac{{\cos }^{2}\,\theta }{2\gamma +{\cos }^{2}\,\theta })$$

where *δϕ* is the acute angle between the two intersecting fibrils (i.e., a value between 0 and *π*/2 radians). Therefore, for a fixed value of *γ* and the estimated *δϕ*, the value of *θ* for each voxel is calculated from the measured value of *κ*_*i*_. In summary, with a rotating linearly polarized input beam focused onto the sample, we detect both the phase and amplitude of the second harmonic signal at two modulation frequencies with lock-in amplifiers; from these data we are able to derive values for the in-plane orientation angle and the out-of-plane orientation angle, providing us with sufficient data to construct 3-dimensional images of collagenous arrays.

### Selection of Tissue to Demonstrate SHARP Capabilities

**Figure 2:**
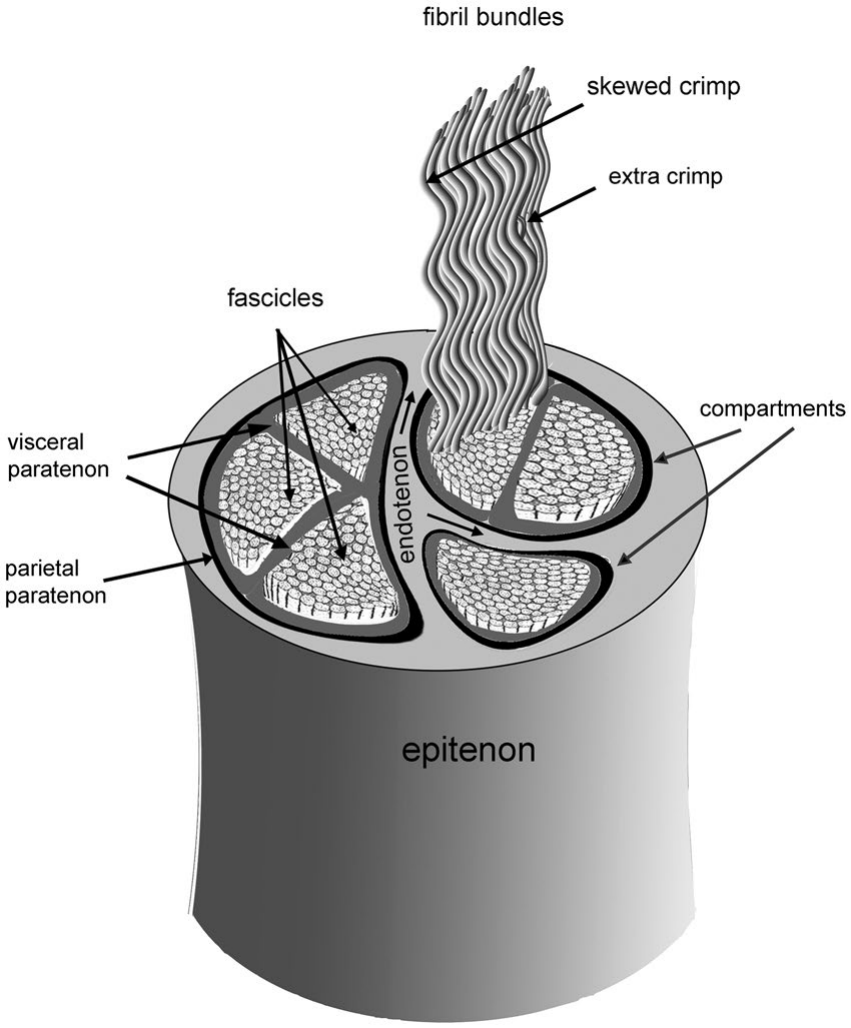
Rat tail tendon morphology: The outer epitenon is a dense interwoven sheath from which arise endotenons, which divide the interior of the tendon into compartments, each lined with a thin sheath, the parietal paratenon. Within each compartment are one or more fascicles, which consist of fibril bundles enclosed within a thin sheath called the visceral paratenon. Legend: light grey: endotenon, black: parietal paratenon, dark grey: visceral paratenon.

SHARP was evaluated using rat tail tendon, as it is a tissue with a strong SHG signal and structurally diverse components, representative of those found in other tissues, which are characterized by complex spatial relationships at many length scales^20–23^. Rat tail tendons are essentially cylindrical tubes on either side of the tail midline, serving as conduits for the Type I collagen fibril bundles that transmit tensile forces between muscle fibers and skeletal elements within the tail^22^. The outer tendon wall, or epitenon, is a woven sheath of type I collagen fibrils. The tendon interior is divided into two or more compartments, whose walls, called endotenons, are extensions of the epitenon (see Fig. 2). Each compartment, lined with a thin collagen layer called the parietal paratenon, contains type I collagen fibrils, which are further subdivided into discrete groups called fascicles. The fibrils within a fascicle are enclosed within a delicate collagenous sheath,the visceral paratenon, whose smooth surface allows fasicles to slide up and down within their compartments in response to external forces. Fascicle mobility is directly related to its ability to transmit tensile forces^22^. Rat tail tendon morphology is not uniform along the length of the tail; the size, shape, and number of compartments varies, as does the number of fascicles. Similarly, the organization of the fibrillar bundles in the fascicles is also heterogeneous^23^, with some fascicles displaying skewed crimp, bundle dislocations, and extra crimp (see Fig. 2). We analyzed both cross-sections and longitudinal sections of rat tail tendon to facilitate comparison with previous reports, including 2-dimensional SHG scans.

### Cross-section of Rat Tail Tendon

**Figure 3:**
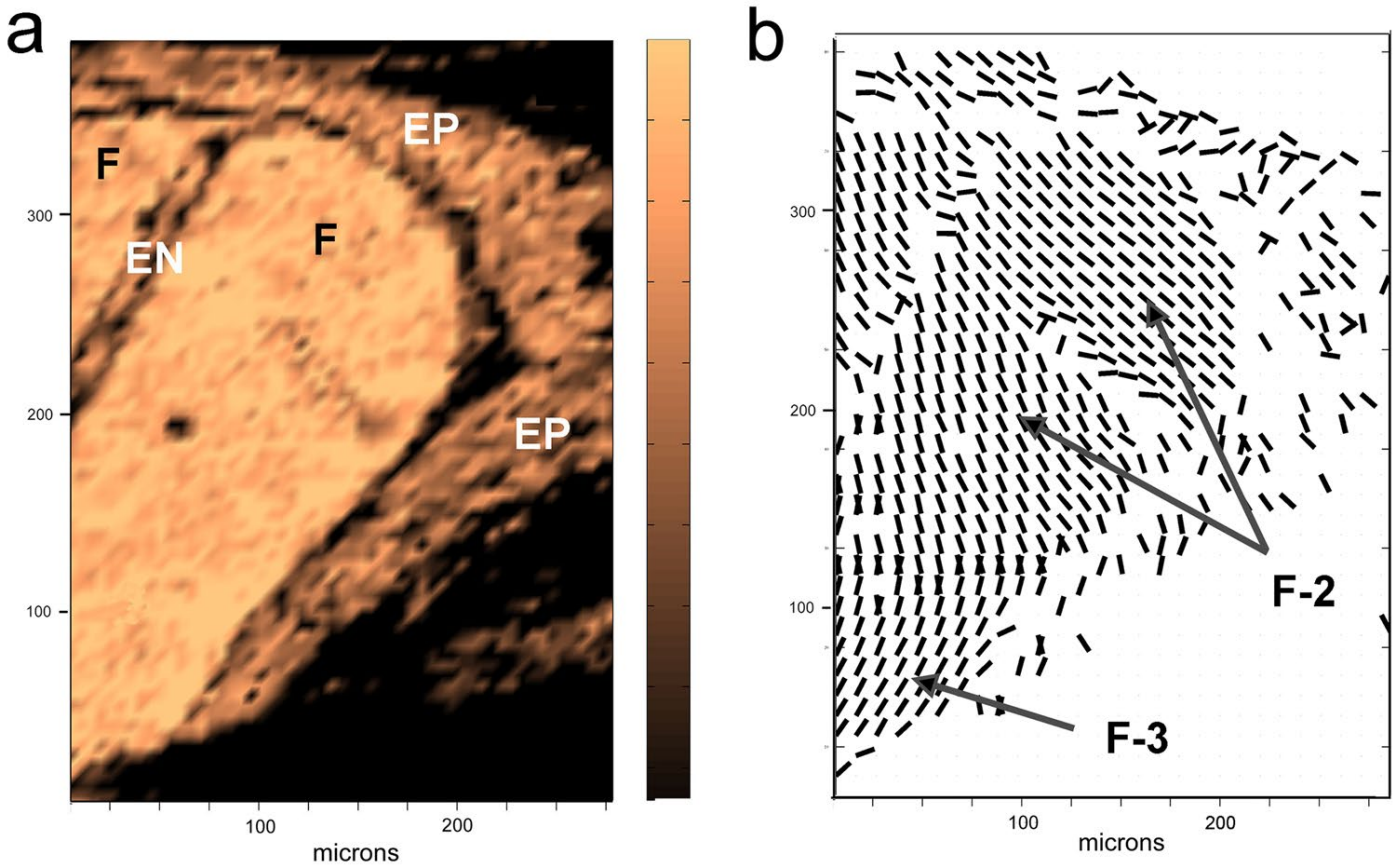
2-D SHG images of a cross-section of rat tail tendon: (**a**) SHG intensity scan of part of a tendon; the epitenon is seen surrounding two compartments, which are separated by an endotenon; (**b**) in-plane orientation data (values for *ϕ*) for the same region, visualized as a vector field. These data indicate that the lower compartment has at least two fascicles, based on the abrupt change in the in-plane orientation of the fibrillar bundles; the upper fascicle (F-2) has an apparent disjunction in the fibrillar bundle organization. Legend: EN = endotenon, EP = epitenon, F = fascicle.

A 2-dimensional SHG scan of a tendon cross-section is shown in Fig. 3, corresponding to the conventional type of section used to illustrate organization of the axially oriented fibrillar bundles. Several structural features can be identified in the 2-dimensional SHG intensity scan shown in Fig. 3a: the outer epitenon with interwoven fibrillar bundles encloses two compartments, separated by an endotenon. The number of fascicles in the compartments cannot be determined from the intensity image. In-plane orientation data, displayed in Fig. 3b as a vector map, provides additional information: Based on the abrupt change in fibrillar orientation in the lower left region of the compartment, there appear to be at least two fascicles present.

**Figure 4:**
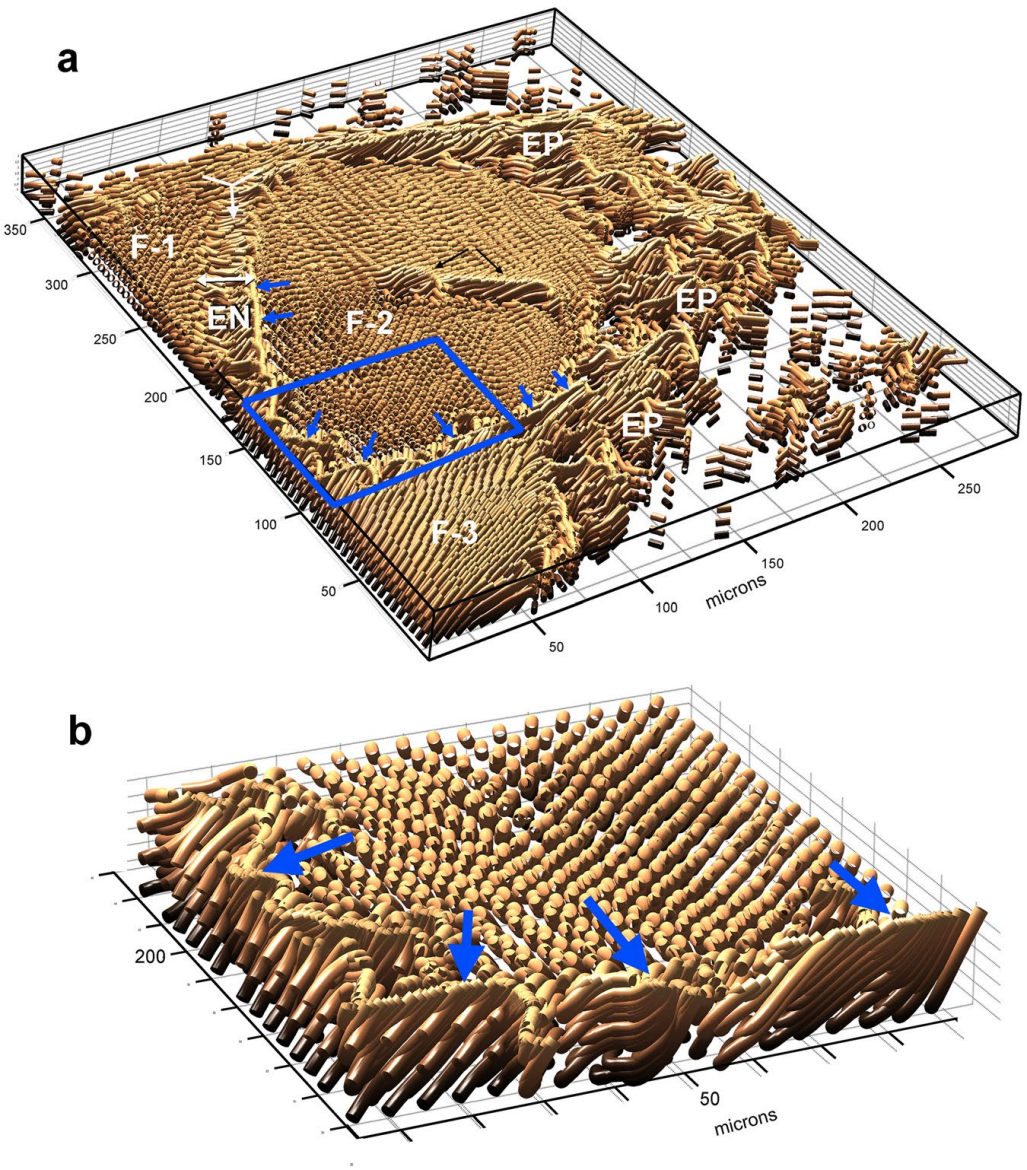
SHARP image of tendon cross-section, rendered using both in-plane and out-of-plane orientation data: (**a**) SHARP image of the sample in Fig. 3 clearly reveals the morphological differences between the ordered fascicles and the woven pattern of the epitenon and endotenon. The presence of two fascicles in the lower compartment is confirmed by the presence of the visceral paratenon, indicated by blue arrows, that separates them. The collagen bundles in the largest fascicle (F-2) show a gradual change in out-of-plane orientation, an indication of skewed crimp; (**b**) Detailed view of the visceral paratenon (see blue box in panel a), characterized by axially oriented, parallel fibrils that form a very thin wall enclosing the fascicles. Legend: EN = endotenon, EP = epitenon, VP = visceral paratenon, F-1, F-2, F-3 = three separate fascicles.

SHARP imaging of the same sample, in which both in-plane and out-of-plane angles were measured, is shown in Fig. 4a. The epitenon, endotenon, and the fascicular regions seen in the the 2- dimensional scan are easily identified, as well as several additional structural details. Note that SHARP imaging is capable of detecting the visceral paratenon (indicated by small blue arrows), which separates the two fascicles in the lower compartment. This structure is essentially a very thin collagenous sheet, viewed end-on in this section, that is frequently undetected in light microscopy^23^. It is identifiable in the SHARP image by its distinctive geometry relative to the surrounding fibrillar bundles, thus underscoring the value of direct detection of orientation angles in three dimensions. We can also see that the fibrillar bundles in the upper fascicle show a gradual increase in their out-of-plane orientation from top to bottom, consistent with the presence of the skewed crimp pattern illustrated in Fig. 2. Structural discontinuity in the middle of the large fascicle (F-2) is clearly visible, consisting of a small group of fibrils with out-of-plane orientation greater than that of neighboring fibrils and a relatively sharp twist in the in-plane angle, suggesting the presence of a bundle dislocation and/or an extra crimp. Figure 4b presents a close-up view of the visceral paratenon that illustrates its morphology more clearly, since only the fasicles lying behind it (in F-2) are included in the image. The paratenon consists of a row of parallel, almost vertical fibrils, lined up next to each other, forming a distinct wall-like structure between the fascicles. Note a slight curvature in several of the paratenon fibrils, consistent with the presence of a very low amplitude crimp, as described by Rowe^22^.

### Longitudinal Section of Rat Tail Tendon

**Figure 5:**
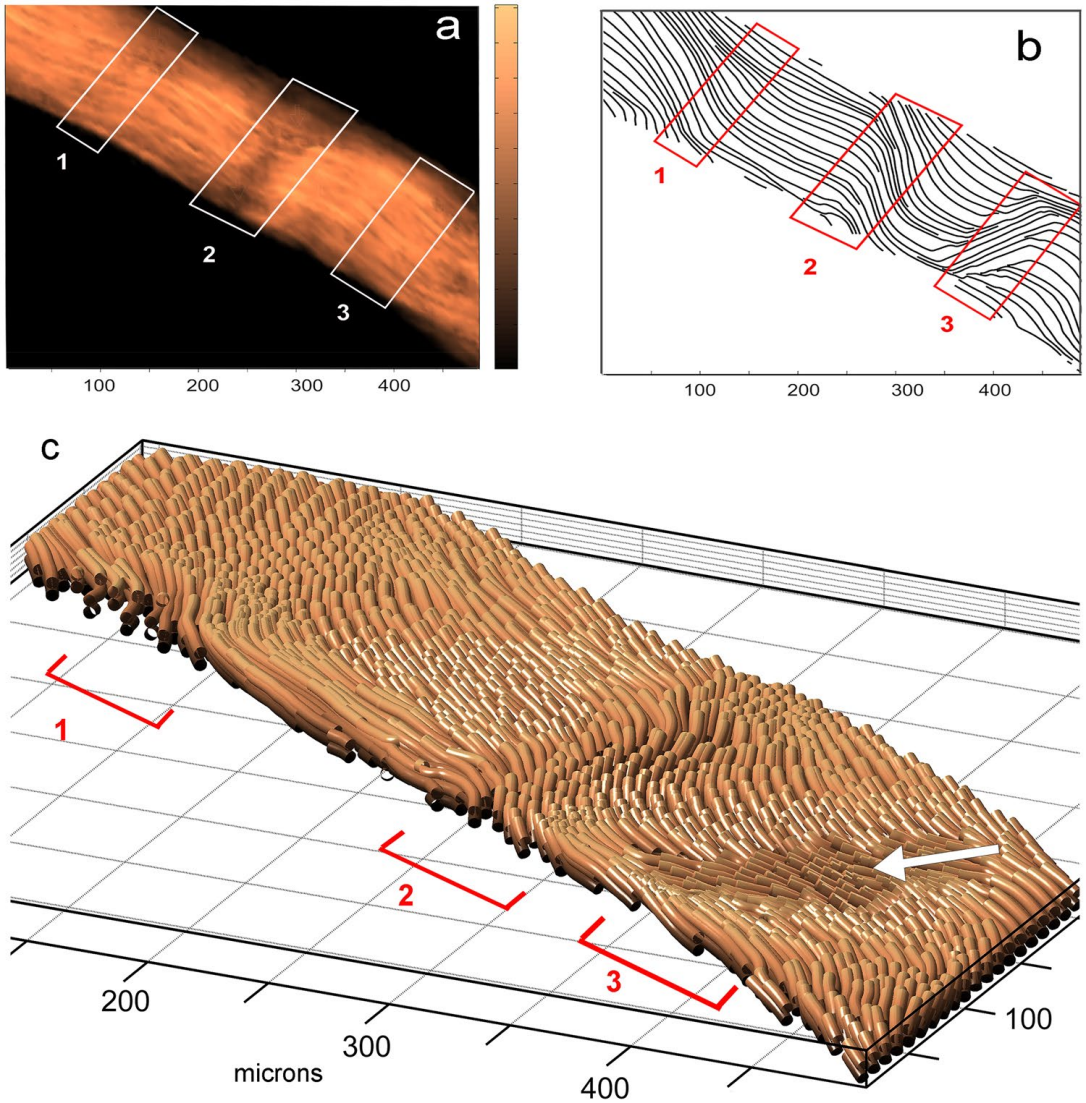
Comparison of 2-D SHG images with SHARP images in a longitudinal section of RTT: (**a**) 2-D SHG intensity scan; the white boxes correspond to regions of collagen crimp identified from orientation data; (**b**) 2-D in-plane orientation angles (*ϕ*), displayed as streamlines; the red boxes correspond to regions of collagen crimp; (**c**) SHARP image of the same RTT sample; the red brackets indicate the same regions as the boxes in the 2-D images; the white arrow indicates the fibrillar bundle seen in box 3 in panel b.

Two-dimensional SHG scans of a longitudinal section of rat tail tendon are shown in Fig. 5a,b. The in-plane orientation data are displayed as streamlines to facilitate visualization of collagen crimp. The putative crimp regions are indicated by numbered boxes in Fig. 5a,b. The 3-dimensional SHARP image is shown in Fig. 5c. Regions 1 and 2 show an undulation pattern similar to that seen in boxes 1 and 2 of the 2-D scans, consistent with the presence of crimp. In contrast, region 3 in the SHARP image shows a discrete fibril bundle (indicated with a white arrow), oriented out and upwards at a fairly sharp angle relative to the neighboring bundles, which is correlated with the sharp downward streamline angle seen in Fig. 5b, box 3. Based on the SHARP image, it seems likely that, in contrast to the crimp pattern in regions 1 and 2, region 3 represents one of the inhomogeneities in fibrillar organization described above.

### SHARP Imaging of Bovine Fascia

**Figure 6:**
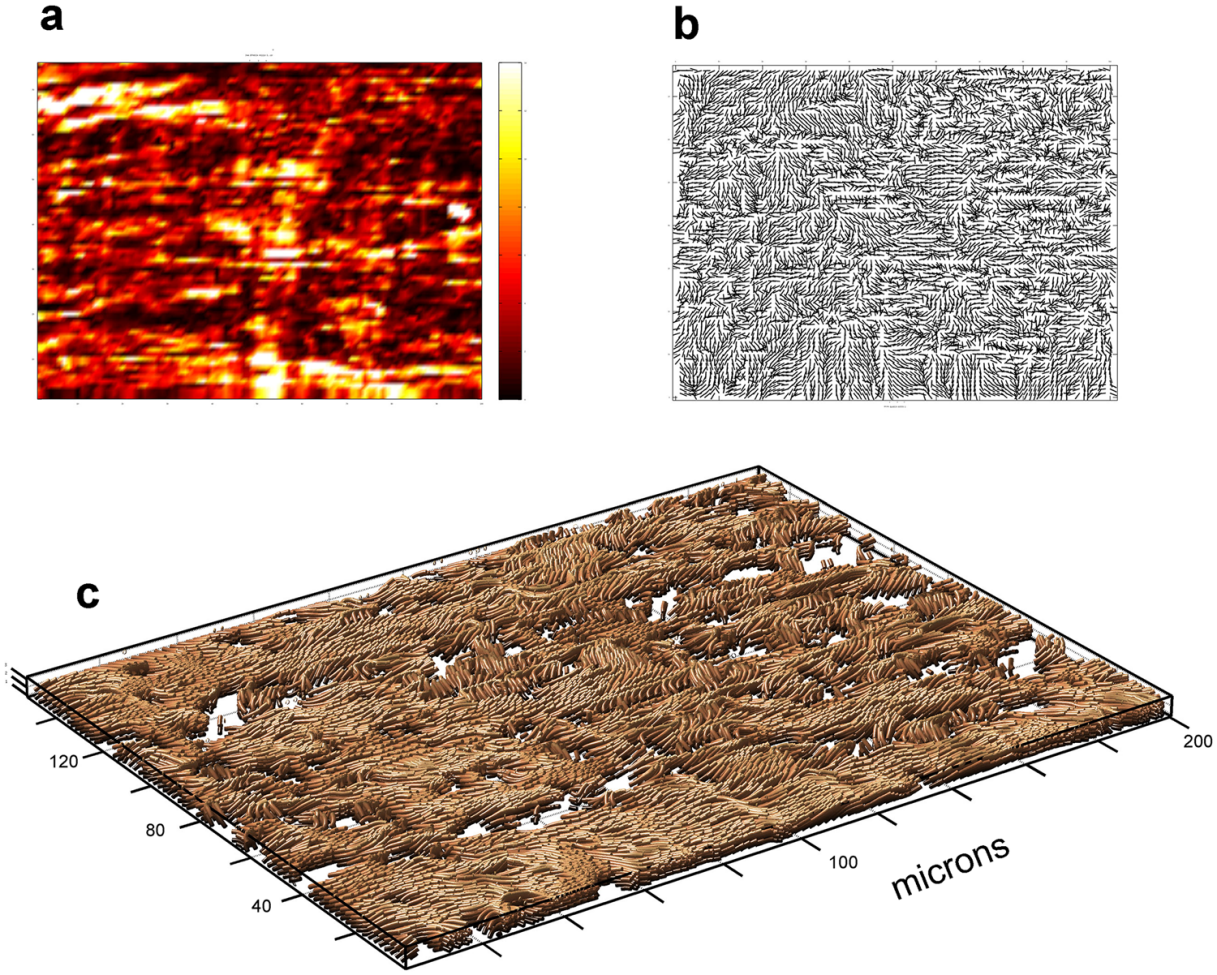
Comparison of 2-D SHG images with SHARP image of bovine deep fascia: (**a**) 2-D SHG intensity image of fascia reveals pattern of transverse fibrillar bundles, interspersed with shorter bundles with varying orientations; (**b**) 2-D in-plane orientation map suggests fibrillar interweaving, but this pattern may also be associated with disorder; (**c**) SHARP image illustrates the relationships among the bundles more clearly.

Fascia represents another tissue with a strong SHG signal^24^, comprised of Type I collagen fibrillar bundles. However, in contrast to rat tail tendon, there is a lack of hierarchical organization and discrete structures. Deep fascia is composed of two distinct layer; the one closest to the muscle is characterized by fairly randomly oriented fibers and fiber bundles. The second layer has distinct, parallel collagenous bundles. Bundles range in size from 2 to over 30 microns in diameter, considerably larger than those in rat tail tendon. The interactions between these two layers are poorly understood, especially in response to strain or injury. In-plane 2D SHG intensity and orientation scans are shown in Fig. 6a,b. In the intensity scan a distinct pattern of parallel fibrillar bundles running transversely can be seen, interspersed with shorter, randomly oriented bundles. The in-plane orientation data seen in Fig. 6b suggest the presence of an interwoven pattern. Structural relationships among the bundles cannot be easily inferred from the 2-dimensional images. In contrast, the SHARP image in Fig. 6c clearly shows both the characteristic layer of parallel bundles with interspersed shorter fibrillar bundles oriented at varying angles to them. *In vitro* studies of bundle interactions under biomechanical loading could be useful in gaining insight into the pathogenesis of fascial dysfunction.

## Discussion

SHARP represents a novel approach for determining the spatial geometry of collagen from the second harmonic signal; to our knowledge it is the first method in which in-plane and out-of-plane orientation angles are directly calculated from modulated polarimetry data obtained in a single scan. Below we review other methods for calculating out-of-plane orientation values using SHG polarimetry that have been reported in the literature.

Several studies have been based on the theoretical calculations of Erikson^25^, describing the relationships among the parallel and perpendicular components of SHG signal intensity, the polarization angle of the incident laser relative to the fibril sample, and the out-of-plane angle of the sample. It is assumed that the ratio of the intensity components is equivalent to the ratio of the squares of the two independent tensor elements in collagen. Generally multiple scans are performed to determine the parallel and perpendicular components of the SHG signal; Erikson’s equations,or variations of them, are then used to calculate the out-of-plane orientation angle on a per pixel basis. This approach has been used to visualize spatial relationships in a variety of tissues, including tendon, skin and cornea^26–28^.

PIPO microscopy represents another method that has been used to determine in-plane and out-of-plane orientation of collagen, among other NLO parameters^9^; this method consists of measuring SHG intensity in 2 × 2 pixel regions using multiple input/output settings. The data are then fit to a general intensity equation using a custom Levenberg-Marquardt algorithm to determine the tensor element ratios, in-plane orientation angles, and an asymmetry parameter, from which nonuniformity of fibril organization is inferred. Fibril orientation is also determined indirectly from diagonal translation of the intensity pattern in the 4-pixel plots. Out-of-plane orientation data can also be extracted under certain constraints. A 3-dimensional rendering of lamellar rings based on data obtained by PIPO has been reported.

More recently, another method capable of determining in-plane and out-of-plane orientation has been reported, based on nonlinear optical Stokes ellipsometry (NOSE)^10, 11^. NOSE is capable of producing 10 unique elliptical polarizations as well as several post-optical processing procedures. In-plane and out-of-plane orientation angles have been reported for several different tissues using this approach. The orientation angles are determined by iterative solutions for global tensor elements and local sample orientation, using an initial guess value and image texture analysis. Results for the out-of-plane angles were displayed in histograms comparing three different tissues; no 3-dimensional visualizations of the tissues were reported.

In comparison with the methods described above, we believe that SHARP offers several advantages. Sufficient data for 3-dimensional visualization are obtained from only a single scan, avoiding potential problems in multi-scan replicability. The orientation angle values are calculated directly from the polarimetry data using an analytical expression,avoiding potential problems associated with optimization and/or fitting algorithms, such as choosing correct initial conditions and fitting parameters. Macroscopic tensor components, experimentally determined and validated, are used for calculating out-of-plane values, avoiding the need to rely on *ab initio* calculations of hyperpolarizability tensor elements or on derivation of molecular tensor ratios from imaging data using fitting algorithms. In summary, SHARP represents a novel, fast method for analyzing 3-dimensional collagen geometry from polarimetry data–in a very real sense, it serves as a direct connection to the information in the specific sample being analyzed, without post-optical or computational intermediaries.

## Methods

### Microscopy Setup

We acquired second harmonic generation data using a custom-built optical setup. A Ti-sapphire laser (Mira, Coherent Inc) was used to generate linearly polarized 200 fs pulses with a wavelength of 800 nm and a repetition rate of 76 MHz; maximum energy was 5 nJ. The beam passed through a half-wave plate, which was rotated to control the power incident on the sample, followed by a polarizing beam splitter and an electrooptic modulator (360–80: Conoptics, Danbury, CT). A function generator (DS345, SRI, Sunnyvale, CA) was used to produce a sawtooth waveform at 4 kHz to drive the EOM voltage such that a linearly polarized beam is produced and the angle of the linear polarization of the beam incident on the sample varies from 0 to 180°. A 50 mm lens then focussed the beam on a 50 micron pinhole, after which it passed through a stationary quarter-wave plate, converting the elliptically polarized light into linearly polarized light. The beam was focussed on the sample with a 20X Mitutoyo Plan Apo Infinity Corrected objective (NA = 0.42, focal length = 10 mm). We used an objective with a relatively low numerical aperture so that the polarization state of the laser beam was not substantially altered in the focus. In case of polarization distortion caused by a larger NA objective, a Babinet-Soheil compensator can be added before the larger NA objective (i.e. NA = 0.8 or smaller) to ensure the paraxial approximation is maintained and the beam is linearly polarized in the focal volume. The transmitted signal was collected with a 100X Mitutoyo Plan Apo Infinity Corrected objective (NA = 0.7, focal length = 2 mm); a dielectric filter allowed only the second harmonic signal to reach the photomultiplier tube (H6780; Hamamatsu Photonics K.K., Hamamatsu City, Japan). The polarization-modulated incident beam generates second harmonic signals in the sample that are modulated at the first and second tone of the modulation frequency of the EOM. After passing through a current preamplifier (SR570, Stanford Research Systems), the signal from the PMT was processed by two lock-in amplifiers (EG & G 7260, Perkin Elmer) set to detect the amplitude and phase at both the first and second harmonic of the EOM frequency. The sample was mounted on an *xyz* translation stage; the scan size in the *xy* plane and the distance between data acquisition points were implemented with a computer-controlled motion controller (ESP300, Newport, Irvine, CA). A Labview program was used to coordinate the motion controller and data acquisition from the two lock-ins for each scan. While we used mechanical stages in this work that do not provide fast scanning, the maximum image acquisition rate of the experimental technique is limited by the bandwidth of the lock-in amplifiers. We have found that a time constant on the order of a few milliseconds achieves acceptable signal-to-noise ratio at each pixel. This is comparable to the 1 ms average pixel dwell time of other SHG image systems^11^.

### Derivation of Equations 1 and 2

In the general case, when the fibril lies out of the plane of polarization, the unit vector along the symmetry axis of the collagen fibril is $\hat{s}={s}_{x}\hat{x}+{s}_{y}\hat{y}+{s}_{z}\hat{z}=\,\cos \,(\theta )\,\sin \,(\varphi )\hat{x}+\,\cos \,(\theta )$ $\cos \,(\varphi )\hat{y}+\,\sin \,(\theta )\hat{z}$. The plane of polarization is the xy-plane, the direction of propagation is $\hat{k}=\hat{z}$ and the unit vector along the electric field is $\hat{e}={e}_{x}\hat{x}+{e}_{y}\hat{y}=\,\cos \,(\alpha )\hat{x}+\,\sin \,(\alpha )\hat{y}$. The vector components of the director parallel to induced polarization $\vec{P}(2\omega )$ is ${p}_{x}={s}_{x}{({s}_{x}{e}_{x}+{s}_{y}{e}_{y})}^{2}+\gamma ({e}_{x}^{2}+{e}_{y}^{2}){s}_{x}+2\gamma ({s}_{x}{e}_{x}+{s}_{y}{e}_{y}){e}_{x}$,and ${p}_{y}={s}_{y}{({s}_{x}{e}_{x}+{s}_{y}{e}_{y})}^{2}+\gamma ({e}_{x}^{2}+{e}_{y}^{2}){s}_{y}+2\gamma ({s}_{x}{e}_{x}+{s}_{y}{e}_{y}){e}_{y}$. The component of the SHG generated along the direction of propagation does not contribute to final SHG intensity to a significant degree (i.e. ignore *p*_*z*_). We express second harmonic intensity in relation to the quantities $\vec{p}$, $\hat{s}$ and $\hat{k}$ as a proportionality, since ratios of the quantities will be taken that will cancel out common factors: ${I}_{SHG}\propto {(\vec{p}\cdot \hat{s})}^{2}+{(\vec{p}\cdot (\hat{s}\times \hat{k}))}^{2}$, from which follows ${I}_{SHG}\propto ({p}_{x}^{2}+{p}_{y}^{2})({s}_{x}^{2}+{s}_{y}^{2})$, which reduces to

4 $${I}_{SHG}\,\alpha \,{I}_{mod0}(\gamma ,\theta )+{I}_{mod1}(\gamma ,\theta )\,\cos \,(2\alpha +2\varphi )+{I}_{mod2}(\gamma ,\theta )\,\cos \,(4\alpha +4\varphi )$$

where

$${I}_{mod1}(\gamma ,\theta )=-\,{\cos }^{4}\,\theta \,({\cos }^{2}\,\theta +2\gamma )({\cos }^{2}\,\theta +4\gamma )/2$$

and

$${I}_{mod2}(\gamma ,\theta )={\cos }^{6}\,\theta \,({\cos }^{2}\,\theta +4\gamma )/8,$$

and the ratio of the *κ* = *I*_*mod*2_/*I*_*mod*1_ reduces to

5 $$\kappa =-\frac{1}{4}(\frac{{\cos }^{2}\,\theta }{2\gamma +{\cos }^{2}\,\theta })$$

### Derivation of Equation 3

Assume the second harmonic is generated by two identical fibrils oriented at some angle with respect to each other. The first fibril generates SHG in 0 < *z* < *l*/2 and the second in the region *l*/2 < *z* < *l*. Then in 0 < *z* < *l* with *E*(2*ω*, *z* = 0) = 0

6 $$E(2\omega ,z=l)=\frac{j\omega E{(\omega )}^{2}}{{n}_{2\omega }}({d}_{eff1}{\int }_{0}^{l\mathrm{/2}}{e}^{-j{\rm{\Delta }}kz}dz+{d}_{eff2}{\int }_{l\mathrm{/2}}^{l}{e}^{-j{\rm{\Delta }}kz}dz)$$

7 $$E(2\omega ,z=l\mathrm{/2})=\frac{j\omega lE{(\omega )}^{2}{e}^{-j3{\rm{\Delta }}kl\mathrm{/4}}}{4{n}_{2\omega }}(\frac{\sin \,({\rm{\Delta }}kl/4)}{{\rm{\Delta }}kl/4})\,({d}_{eff1}{e}^{j{\rm{\Delta }}kl\mathrm{/4}}+{d}_{eff2}{e}^{-j{\rm{\Delta }}kl\mathrm{/4}})$$

where *E* is the electric field, *n*_2_ *ω* is the index of refraction at the second harmonic frequency, and *δ*k is the phase mismatch between the fundamental and second harmonic frequency. This implies that $\vec{P}={\varepsilon }_{o}a{E}_{1}^{2}({\vec{p}}_{1}{e}^{j{\rm{\Delta }}kl\mathrm{/4}}+{\vec{p}}_{2}{e}^{-j{\rm{\Delta }}kl\mathrm{/4}})$. Let the first fibril be oriented with respect to the x axis at an angle *δϕ*/2 and the second fibril at an angle −*δϕ*/2 in the xy-plane. That is, for the first fibril ${\hat{s}}_{1}=\cos \,(\theta )\,\sin \,(\delta \varphi )\hat{x}+\,\cos \,(\theta )\,\cos \,(\delta \varphi )\hat{y}+$ $\sin \,(\theta )\hat{z}$, and the second fibril ${\hat{s}}_{2}=\,\cos \,(\theta )\,\sin \,(\delta \varphi )\hat{x}-\,\cos \,(\theta )\,\cos \,(\delta \varphi )\hat{y}+\,\sin \,(\theta )\hat{z}$. The SHG intensity is

8 $$\begin{array}{l}{I}_{SHG}\,\alpha \frac{1}{4}({p}_{x1}^{2}+{p}_{y1}^{2})({s}_{x1}^{2}+{s}_{y1}^{2})+\frac{1}{4}({p}_{x2}^{2}+{p}_{y2}^{2})({s}_{x2}^{2}+{s}_{y2}^{2})\\ \quad +\frac{1}{2}({\vec{p}}_{1}\cdot {\hat{s}}_{1})({\vec{p}}_{2}\cdot {\hat{s}}_{2})\,\cos \,(\tfrac{\pi l}{2{l}_{c}})\end{array}$$

where *l*_*c*_ = *π*/Δ*k* is the coherence length, ${l}_{c}\approx 7\mu m$ for collagen^13^. The last term is the interference contribution between the electric fields between the two fibrils, and will be negligible if interaction length is approximately equal to *l*_*c*_ (typically the case in experiments). Thus, substituting into Eq. (1) for the in-plane orientation angle $\varphi +\tfrac{1}{2}\delta \varphi$ for the first fibril and $\varphi -\tfrac{1}{2}\delta \varphi$ for the second, it follows

$$\begin{array}{lll}{I}_{SHG} & \propto  & {I}_{mod0}(\gamma )+{I}_{mod1}(\gamma )\,\cos \,(\delta \varphi )\,\cos \,(2\alpha +2\varphi )\\  &  & +{I}_{mod2}(\gamma )\,\cos \,(2\delta \varphi )\,\cos \,(4\alpha +4\varphi )\end{array}$$

and finally

9 $${\kappa }_{i}=\frac{\cos \,(2\delta \varphi )}{\cos \,(\delta \varphi )}\kappa $$

### Detection of intersecting fibrils

Many tissues contain structures with differing in-plane orientations. Voxels located at the boundary between such regions may contain fibrils from both regions, which, being non-parallel, will intersect^29^. It is assumed that no more than two such groups are likely to be present in a given voxel, and that each group contributes equally to the SHG intensity^6, 13, 30^. Using sliding nearest neighbor analysis of in-plane orientation angle data (*ϕ*), boundary voxels are identified if the mean difference between the index voxel *ϕ* value and values of its 8 nearest neighbors is greater than 10°^?^. To determine if the boundary voxel contains intersecting fibrils, mean *ϕ* values are calculated for the 6 pairs of voxels surrounding each index boundary voxel. If the mean value of a voxel pair is within 10° of the index voxel value for *ϕ*, it is assumed that the index voxel contains the two fibrils from the voxel pair. *δϕ* is defined as the acute angle between these two fibrils. This method was validated using mouse annulus fibrosus, in which the lamellar rings represent a series of discrete regions whose boundaries are characterized by abrupt changes in fibrillar orientations. Photomicrographs of the annulus fibrosus under polarized light confirmed that fibrillar orientation alternated between adjacent lamellae with no evidence of disruption in the lamellar boundaries. Boundary voxels between neighboring lamellae were identified using nearest neighbor analysis of in-plane orientation data; among the boundary voxels, those identified as containing intersecting fibrils corresponded to a discontinuity in the sharp boundary between the adjacent lamellae displayed in the map of *ϕ* values.
